# Reassessing the Role of Intra-Arterial Drug Delivery for Glioblastoma Multiforme Treatment

**DOI:** 10.1155/2015/405735

**Published:** 2015-12-30

**Authors:** Jason A. Ellis, Matei Banu, Shaolie S. Hossain, Rajinder Singh-Moon, Sean D. Lavine, Jeffrey N. Bruce, Shailendra Joshi

**Affiliations:** ^1^Department of Neurological Surgery, Columbia University Medical Center, New York, NY 10032, USA; ^2^Department of Molecular Cardiology, Texas Heart Institute, Houston, TX 77030, USA; ^3^School of Engineering and Applied Science, Columbia University, New York, NY 10032, USA; ^4^Department of Anesthesiology, Columbia University Medical Center, New York, NY 10032, USA

## Abstract

Effective treatment for glioblastoma (GBM) will likely require targeted delivery of several specific pharmacological agents simultaneously. Intra-arterial (IA) delivery is one technique for targeting the tumor site with multiple agents. Although IA chemotherapy for glioblastoma (GBM) has been attempted since the 1950s, the predicted benefits remain unproven in clinical practice. This review focuses on innovative approaches to IA drug delivery in treating GBM. Guided by novel* in vitro* and* in vivo* optical measurements, newer pharmacokinetic models promise to better define the complex relationship between background cerebral blood flow and drug injection parameters. Advanced optical technologies and tracers, unique nanoparticles designs, new cellular targets, and rational drug formulations are continuously modifying the therapeutic landscape for GBM. Personalized treatment approaches are emerging; however, such tailored approaches will largely depend on effective drug delivery techniques and on the ability to simultaneously deliver multidrug regimens. These new paradigms for tumor-selective drug delivery herald dramatic improvements in the effectiveness of IA chemotherapy for GBM. Therefore, within this context of so-called “precision medicine,” the role of IA delivery for GBM is thoroughly reassessed.

## 1. Introduction

Although drugs directed against glioblastoma may be delivered by a number of routes, from a physiological standpoint, intra-arterial (IA) drug delivery is an appealing method (Table 1) [1]. Drugs are distributed through the capillary network within a narrow volume of distribution, physically restricted by local factors. Tissue drug perfusion is theoretically very efficient following a path of nutrient diffusion [2]. However, the pharmacokinetics of IA drug delivery is exceedingly complex [3–6]. For effective IA drug delivery, drugs have to be rapidly and, preferably, irreversibly taken up during their first pass through the tissue circulation, lasting between 1 and 10 seconds in the brain [7–9]. Therefore, IA pharmacokinetics requires an understanding of several important parameters including (1) physiologic and anatomic factors that influence regional blood flow, (2) hydrodynamic factors that affect drug delivery, (3) injection parameters and endothelium-drug interactions, (4) kinetics of uptake and transfer across the blood brain barrier (BBB), and (5) site- or tissue-specific pharmacokinetics [3, 6, 10–13].

The lack of robust experimental and theoretical models, leading to inadequate optimization of injection parameters as well as a lack of rationalization in drug selection, further hinders improvements in IA drug delivery [5, 14–16]. However, IA drugs have been widely used in recent years, either off-label or as part of clinical trials [14, 17–26]. Most of these attempts rely on the general belief that local injections, transiently generating high arterial blood concentrations, will lead to the desired pharmacodynamic effects. Poor rationalization and selection for IA interventions could lead to treatment failure as well as significant adverse events. This is even more disconcerting since IA interventions are generally performed in extremis, in recurrent or even end-stage cases, with a severely skewed risk-benefit ratio.

IA anesthetic drugs have been used to localize brain functions since 1948, providing a robust platform for the safe use of this delivery method [27]. Attempts to treat GBM with IA chemotherapy began soon after the Second World War, with no significant impact on outcomes [28]. On the other hand, IA chemotherapy is now routinely used for the treatment of retinoblastoma and advanced liver cancer, improving quality of life and extending overall survival [29–33]. Beyond these two well-established applications, IA chemotherapy has been also used, with variable success, in the treatment of other cancers such as breast cancer, head and neck cancer, colorectal cancer, penile cancer, and pancreatic cancer [34–39].

The failure of previous IA chemotherapies to effectively treat GBM is not unique, however. Numerous other treatment modalities have had little impact on the clinical course of this disease. Several recent clinical trials have or currently are assessing IA drugs for GBM treatment (Table 2). Given the failure to affect outcomes over the last several decades, the question arises whether one should persist in evaluating IA chemotherapy for brain tumor treatment. In light of recent technological and therapeutic advances in GBM treatment, what is the role of IA therapy?

The following review supports revisiting IA drug delivery for the treatment of GBM. Emerging optical technologies offer novel insight into the complex pharmacokinetics of IA drug delivery and may lead to improved clinical effectiveness [40–43]. Concurrent reduction of blood flow appears to enhance the regional effectiveness of IA drug delivery [41, 42, 44]. New tumor targeting strategies using computational analysis of phenotypic and genotypic characteristics as well as nanodelivery platforms may further improve GBM treatment [45]. Thus, we believe that the insights provided by these novel technologies will improve drug targeting while significantly minimizing complications that have plagued the field in the past.

## 2. Brief History

Warner Frossman's development of cardiac catheterization was driven, in part, by the hope of locally targeting drug treatments for heart failure. His concept of local pharmacological intervention using catheters appealed to many at the time [46–48]. The interest in IA drugs received a significant boost in 1948, after Wada demonstrated the effectiveness of local anesthetic drug delivery in generating cortical electrical quiescence [27, 49]. The Wada test for localization of neurological functions has been used virtually unchanged over the past sixty years [50]. Targeted IA chemotherapy was first used in the years following the Second World War [51]. Calvin Klopp at George Washington University used IA chemotherapy for head and neck tumors as well as glioblastoma, in 1950 [28, 52]. In the 1960s, Charles Wilson systematically investigated IA chemotherapy for glioma treatment [53–55]. In 1972, Stanley Rapport demonstrated hyperosmotic disruption of the BBB [56, 57]. Significant advances were made at the NIH by Oldfield and others in the 1980s [15, 58–60]. Most IA therapies focused on nitrogen mustards; their high lipid solubility and rapid onset of action made these drugs particularly appealing for IA administration [61]. However, unexplained neurotoxicity, in particular white matter infarction, emerged as serious complications with IA nitrogen mustards, thereby limiting their usage [62–65]. By the 1990s, interest in IA chemotherapy for brain tumors started to wane. Edward Neuwelt was a fervent advocate of IA chemotherapy throughout the 1990s, incorporating it into his BBB disruption program [66, 67]. Unfortunately, the loss of drive to further advance IA therapeutics for GBM and other brain neoplasms could not have been more untimely. Modern endovascular technology was rapidly advanced in the 1990s and continues to make significant technological strides. Furthermore, the past decade has seen advances in optical imaging and nanoparticle engineering, both with the potential to impact IA drug delivery immensely.

In sharp contrast to the treatment of GBM, interest in IA chemotherapy as a treatment modality for certain other malignancies has been steadily increasing. Reese et al. first used IA chemotherapy for the treatment of retinoblastoma in 1954 [68]. Today, IA chemotherapy is widely used as a treatment for this tumor. Data demonstrates that IA chemotherapy extends organ survival, avoids disfiguring ablative surgery, and may promote functional recovery [32, 69–71]. Sullivan et al. introduced hepatic artery (HA) chemotherapy in 1964 [72]. HA chemotherapy via implanted infusion pumps is now routinely used for the treatment of unresectable hepatic metastases [73, 74]. In the 1980s, complete breast tumor remission was reported following internal mammary artery infusions of chemotherapy drugs over several days [75]. IA chemotherapy is currently being used in cases of locally advanced breast cancer [39, 76, 77].

Why has IA chemotherapy been so successful in other organs but not in the brain? The challenges for GBM treatment stem from the unique anatomical and physiological characteristics of the brain. Characteristics of the brain including its high resting blood flow, its susceptibility to embolic injury, and its relative environmental isolation provided by the BBB increase the risks and challenges associated with IA drug delivery. However, with safer drugs, advanced imaging methods, and modern endovascular techniques, these hurdles to IA treatment for GBM are not insurmountable.

## 3. Challenges to IA Drug Delivery for GBM

### 3.1. Biological Hurdles

Critical examination of GBM tissue reveals numerous impediments to its treatment with any single modality. GBM is heterogeneous, at both the molecular-genetic and the cellular-tissue levels [78, 79]. To date, there are no consistent tumor-specific phenotypic or genotypic targets. There is always extensive tissue infiltration preventing complete surgical resection. Following surgery, recurrence is universal and generally occurs at the surgical margins. Extensive areas of tumor infiltration, necrosis, hemorrhage, and thrombosis impede effective drug delivery. Certain regions of the tumor are hypoperfused due to ineffective tumor angiogenesis, while others are highly vascular [80]. Given this structural variability, it is likely that drug penetration is variable in different regions of the same tumor [81]. Furthermore, certain regions of the tumor might be inaccessible to IA drugs. Although transient radiographic evidence of IA chemotherapy treatment response in high grade glioma has been reported, no studies have shown durable patient benefit [44]. Furthermore, the question of whether clinical improvements can be made with removal of structural impediments to drug delivery by performing surgery remains unanswered.

### 3.2. Clinical Experience

It is estimated that over 2000 patients have been treated with IA chemotherapy for GBM, mostly in the setting of Phases I and II trials [82]. Thus far, there has been little evidence of significantly improved outcomes during these trials. Several series report prolonged survival by a few weeks, with ocular toxicity or neurotoxicity occurring in 7–50% of the patients [82]. These toxicities were more evident with carmustine. In many instances, IA chemotherapy was delivered proximally to the entire hemisphere via intracarotid injections. Subsequently, supraophthalmic injections were used to decrease toxicity.

The clearance of highly diffusible compounds from brain tissue is directly proportional to the regional blood flow [83]. With hemispheric drug infusion, diffusible compounds pose an elevated risk of injury to white matter tracts. Due to lower blood flow in the white matter as compared to gray matter, this may explain the occurrence of white matter lesions with the administration of IA nitrosoureas. Thus, unless drugs are specifically selected and formulated for tumor uptake, the benefits of IA drug delivery will not be fully reaped.

### 3.3. Treatment Goals

Studies on IA chemotherapy differ significantly in drug injection protocols with respect to the site, dose, timing, anesthetic management, and underlying rationale. Due to this lack of consistency, it is often difficult to compare outcomes from various studies. Lack of standardization is, in part, due to our incomplete understanding of the pharmacokinetics of IA drug injections [5, 82]. Although the primary goal of IA drug delivery is to selectively target the tumor tissue, this goal is often difficult to achieve due to a lack of drug-tumor selectivity and the complex angioarchitecture of GBM. Unlike conventional cerebrovascular lesions, such as an arteriovenous malformation, a GBM's vascular supply may arise from adjacent vascular territories or even from the contralateral hemisphere. Such diffuse vascular input impedes selective drug delivery. Therefore, with IA delivery, drugs are often delivered to both the tumor and the neighboring brain tissue. Complications resulting in neurological deterioration, blindness, cognitive impairment, and seizures are often due to iatrogenic brain injury from nontarget delivery. Without a comprehensive tumor targeting strategy, the goal of IA chemotherapy often becomes a quest for safe delivery of systemic drug doses. Gobin et al. used fractionated algorithms to decrease local toxicity [14]. With this approach, the dose of drug is proportional to the regional blood flow. Therefore, the benefits of the high transient arterial blood concentrations have to be combined with pharmacokinetic or pharmacodynamic selectivity to the tumor tissue. The goal of IA treatments should be tumor-selective drug delivery such that local and systemic complications can be mitigated.

### 3.4. Lack of Reliable Pharmacokinetic Models

**Figure 1:**
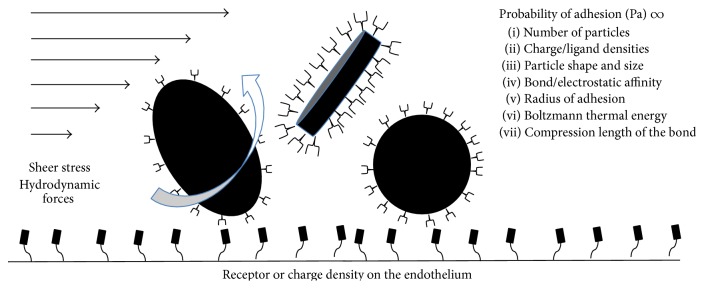
Hydrodynamic forces on drug particles. Shear stress, particle shape, and ligand-receptor interactions are the dominant forces that affect the delivery of particles to their target site.

One of the fundamental problems in translating IA treatment into the clinical setting is the lack of robust pharmacokinetic models. Models used to assess the benefits of IA delivery rely on simplistic concepts and have repeatedly failed to predict tissue drug concentrations [5, 84, 85]. The relevance of these models is further challenged by the evolution of nanotechnology, with larger particles subject to considerably greater hydrodynamic forces [10, 86]. Hydrodynamic forces, as determined by vascular geometry, shear stress, and rate constants of endothelium-drug interactions are critical in determining the probability of the drug to adhere to the endothelium (Figure 1). However, preclinical models thus far have failed to take such parameters into account. Most studies have used snapshot methods to determine tissue drug concentrations at a fixed time point. Technologies for real-time tracking of tissue drug distribution and concentrations, such as PET, were seldom used [87, 88]. Concurrent blood flow measurements are also required for adequate monitoring of drug uptake [89].

In recent years, optical methods have emerged with the capability of tracking tissue tracer concentrations of drugs in subsecond time domains (Table 3) [41, 43, 90–93]. These methods can be combined with complementary techniques to simultaneously assess cerebral blood flow [11, 41, 42, 94–96]. Experimental data tracking tissue concentrations and blood flow changes could generate more accurate pharmacokinetic models that include hydrodynamic factors largely ignored in the past.

### 3.5. Streaming

Cerebral blood flow plays an integral role in IA drug delivery. However, in most clinical studies, parameters such as the cerebral blood flow are not monitored or modified. Some investigators have recommended that blood flow should be increased to improve regional drug delivery. In a series of experiments performed* in vitro* and in primates, Saris and Lutz showed the negative effects of streaming on drug distribution after low volume internal carotid artery (ICA) injections [4, 97, 98]. Streaming was less likely to affect distal injections in the cerebral circulation, for example, after supraophthalmic infusion. Streaming can be decreased by injecting volumes exceeding 20% of the background blood flow rate, by injecting during the diastole, or by injecting using catheters with side ports [4, 97, 99]. It is clear that subtle differences in injection protocols can have profound effects on both the therapeutic response and the occurrence of adverse events. Standardization of delivery protocols and defining concurrent blood flow conditions could have an important impact on the results of clinical studies.

### 3.6. Safety Concerns

IA drug delivery carries risks such as complications related to catheter placement and vascular access, local and systemic complications due to chemotherapeutic drug infusion, and complications due to blood brain barrier disruption. On the other hand, the safety of endovascular procedures has increased tremendously and short-term IA drug delivery is now considered to be generally safe [100, 101]. Local reactions to chemotherapeutic drug infusions, such as ocular erythema with cisplatin, can be minimized by superselective catheter placement upstream of the ophthalmic artery [102]. Although superselective IA chemotherapy can result in streaming, employing specific delivery techniques, as previously described, can mitigate this phenomenon [97]. Selective tumor targeting is perhaps the best way to prevent inadvertent neurological injury and minimize systemic complications.

## 4. Advantages of IA Drug Delivery for GBM

### 4.1. Local Drug Delivery

**Figure 2:**
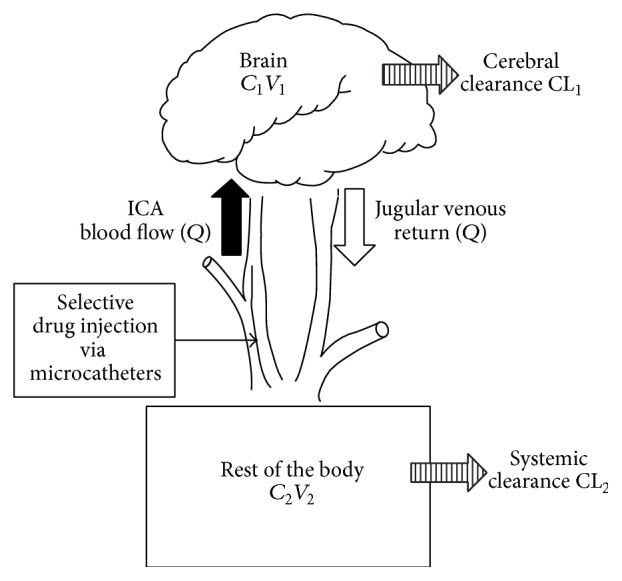
Dedrick model of intracarotid delivery. This pictorial representation of the mathematical model demonstrates that IA drug delivery is most efficient when regional flow (*Q*) is low, regional extraction is high, and systemic clearance is rapid. *C*: concentration, *V*: volume, CL: clearance, and *Q*: regional blood flow.

IA injections rely on drug delivery through capillary networks and eventually into the perfused tissue. The tissue concentrations achieved by IA delivery are considerably higher than those achieved after IV delivery [82, 88]. This high concentration can further increase the tissue diffusion gradient. For highly lipid soluble drugs, such as nitrogen mustards, rapid targeting of tumor tissue is possible [88]. The key to effective IA drug delivery lies in proper drug selection as well as in optimizing the delivery technique based on specific controllable parameters (Figure 2). As discussed later, this can be achieved by increasing the probability that drug molecules attach to the endothelium [10].

### 4.2. Unique Pharmacokinetics

**Figure 3:**
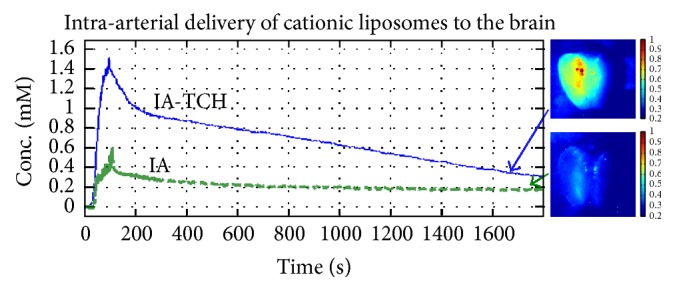
Hypoperfusion-assisted intra-arterial drug delivery. Transient cerebral hypoperfusion (TCH) significantly facilitates the delivery of cationic liposomes to the brain. Multispectral images (MSI) show that cationic liposomal uptake is significantly improved by utilizing intra-arterial delivery with TCH (right panel). Corresponding concentration-time curves obtained by diffuse reflectance spectroscopy quantitatively corroborate these optical phenomena (left panel).

Hydrodynamic factors related to background blood flow, injection characteristics, and vascular geometry play a major role in determining tissue concentrations after IA drug injections. In computational models and in preclinical experiments, reduction of cerebral blood flow improved regional drug delivery [11, 41, 42, 89, 95, 96]. When a bolus of drug is injected during transient cerebral hypoperfusion (TCH), tissue concentrations can be significantly increased (Figure 3). Cerebral hypoperfusion decreases hydrodynamic stress on the injected molecules. It increases drug transit time through the cerebral circulation. Furthermore, it delivers pure drug to the vascular endothelium and decreases opsonization by serum proteins and blood cells.

### 4.3. Dose Advantages

The net advantage of IA drug injection over IV injection depends on a number of factors such as the method of injection, the rate of injection, and the duration of infusions. Comparing IA to IV by using PET measurements in human subjects has revealed a 50-fold increase in tumor tissue concentrations after IA injections [88]. In rabbits, when BCNU concentrations were measured 5 minutes after drug injection, there was a 6-fold improvement in drug delivery [96]. With cationic liposomes, we have observed a similar 50-fold improvement in liposome delivery with IA versus IV injection [42, 95]. We noticed an additional 3- to 10-fold improvement in tissue concentrations by injecting drugs during TCH compared to IA injections without flow arrest.

### 4.4. Systemic Rescue

One of the potential advantages of IA drug delivery is the ability to neutralize the recirculating drug with an antidote or to physically remove drugs by hemoperfusion [15, 58–60]. Such an approach can decrease the chances of systemic side effects. Ototoxicity and nephrotoxicity are known complications of IA cisplatin therapy which can be reduced with concurrent thiosulfate infusion [103, 104]. Trials are underway to evaluate the effectiveness of this combination.

## 5. Investigating the Kinetics of IA Drugs

Critical to the development of the field of IA therapeutics is the understanding of first-pass kinetics during drug delivery. Most conventional methods (see below) do not provide the subsecond time resolution needed to investigate first-pass kinetics. These methods usually do not assess cerebral blood flow and lack adequate spatial resolution. Furthermore, these methods require complicated chemical drug extraction techniques, pose radiation and magnetic hazards, and can be resource- and cost-intensive. However, dramatic advances have been made in optical drug and tracer concentration measurements. Many of these methods can measure tissue drug concentration and blood flow. Provided that the drug has a suitable spectral profile, one can track drug delivery in real-time, map distribution in gross postmortem samples and even track drug and metabolites at a cellular level.

### 5.1. Brain/Plasma Partition Ratio

In this method, the relative concentration of the drug in the brain tissue and plasma is simultaneously determined at a specified time point [105]. The drug remaining in the vascular dead space is not flushed and that amount is therefore included as part of the brain drug concentration. This approach provides a snapshot measurement and is resource-intensive. Additionally, several animals have to be sacrificed to obtain a concentration-time curve.

### 5.2. In Situ Perfusion

**Figure 4:**
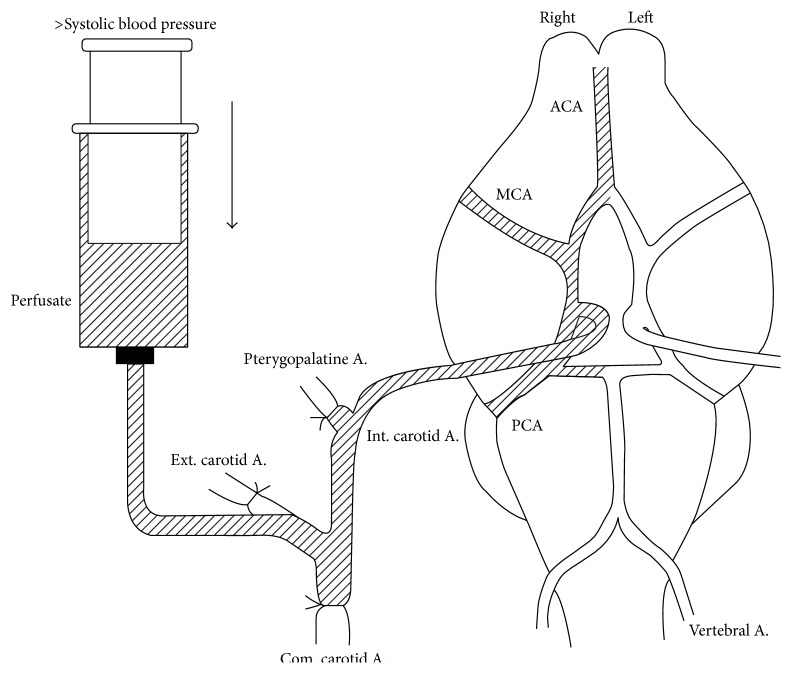
In situ perfusion in an animal model. Used with permission from Takasato et al. [107].

In this method, the internal carotid artery is isolated and drugs are infused over time (Figure 4). The carotid is flushed out and brain tissue drug concentration is determined. There are two pharmacokinetic parameters that describe brain drug uptake: *K*_in_ and PS. *K*_in_ is the net inwards flux of a drug. PS is permeability surface area product; it is the plasma volume that is cleared of a drug during its passage through the cerebral circulation. With the in situ method, *K*_in_ = *C*_br_/(*C*_pf_ × *T*), where *C*_br_ is the concentration of compound in the brain (mg/g), *C*_pf_ is the concentration of compound in the perfusion fluid (mg/mL), and *T* is the time. Using this method, PS can be determined by the Renkin Crone equation: PS = *Q*ln⁡(1 − *K*_in_/*Q*), where *Q* is the cerebral blood flow [106].

### 5.3. Brain Uptake Index (BUI)

The classical method to determine BUI as described by Oldendorf in the 1970s involves injection of the isotope labeled drug into the common carotid artery of a rat [6, 108, 109]. The typical volume is 200 *µ*L and the injection is made over 0.5 s. Fifteen seconds after injection, the animal is sacrificed and the brain tissue is harvested. The drugs are labeled with ^3^H and the uptake of the drug is normalized to the uptake of ^14^C labeled butanol that is freely diffusible into the brain tissue. BUI is then calculated using the following equation: BUI = (^3^H brain/^14^C brain)/(^3^H injection/^14^C injection). BUI is a fraction but can also be expressed as a percentage. BUI represents net uptake of a drug during the first pass through the cerebral circulation [85]. Hardebo and Nilsson have challenged this view, suggesting that recirculation of a drug can occur in 15 s, thereby recommending obtaining tissue samples 5 s after intracarotid injection [6]. BUI provides a good measure for compounds that rapidly diffuse into the brain and are well retained by the brain tissue.

### 5.4. Microdialysis

For intravenous drug delivery with steady state infusion, brain tissue concentrations can be determined by microdialysis. The technique can be used with steady state IA injections as well [110, 111]. However, with bolus IA injections, changes in tissue drug concentration might be too rapid to obtain a sample and injection of the drug could potentially destabilize the probe. Hence, microdialysis is rarely used to investigate the kinetics of IA drugs. The main advantage of microdialysis is its ability to obtain multiple measurements over extended time periods.

### 5.5. Autoradiography

Using radiolabeled drugs, it is possible to obtain a map of drug delivery following IA infusion. The method provides only a single data point per animal. It has been used in both large and small animal models [98, 99, 112].

### 5.6. Positron Emission Tomography (PET)

PET imaging is a very useful tool to investigate the kinetics of IA drugs. This method can track drug delivery in a single animal. However, it is resource-intensive, requires a cyclotron, and has poor spatial resolution [88].

### 5.7. Novel Optical Approaches

The key to the understanding of IA drug kinetics is to be able to track tracer concentrations within a subsecond time frame and to concurrently assess cerebral blood flow either directly or by using an additional device. Newly available optical tools such as diffuse reflectance spectroscopy permit simultaneous measurements of blood flow as well as optical tracer and drug concentrations [113–116]. Optical measurements are possible in a subsecond time frame and are site-specific, and the method is tissue nondestructive. Table 3 summarizes some of the advantages and disadvantages of these novel optical methods. These methods are cost effective and relatively simple to execute once optimized, and they do not carry the hazards of radiation and magnetic fields. Such optical tools can generate pharmacokinetic insights and generate models that could guide future drug development.

## 6. Pharmacokinetics of IA Drugs

Many investigators use simple flow and volume calculations to determine the pharmacokinetic benefits of IA drugs. However, these simple models of IA drug delivery overlook the underlying hydrodynamic complexity. The complex conceptual framework of IA drug delivery requires an understanding of fluid dynamics of both cerebral blood flow and the drug injection. One must simultaneously take into account the rapid first-pass uptake of the drug by the endothelium, transfer across the blood brain barrier, the local release of drug from potential carriers, the tissue pharmacokinetics including local metabolism, and finally the first-pass elimination and recirculation of the drug.

### 6.1. Basic Model of IA Drug Delivery

**Figure 5:**
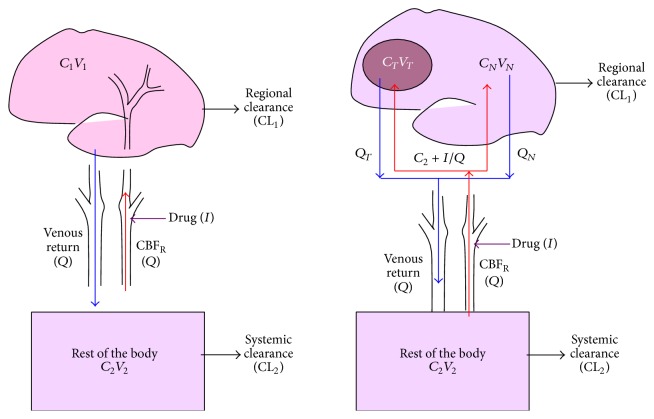
Models of intracarotid drug delivery with and without brain tumor.

The significance of the Dedrick model lies in clearly defining when IA drug delivery truly works (Figure 5) [3]. If the advantage of IA regional drug delivery (Rd) is defined as the (*C*_1_/*C*_2_)_IA_/(*C*_1_/*C*_2_)_IV_, then Rd can be also represented as follows: Rd = 1 + (CL_TB_/*Q* × (1 − *E*)), where CL_TB_ is the total body clearance of the drug, *Q* is the regional blood flow, and *E* is the fraction of drug extracted in the first pass through the cerebral circulation. This basic model has several implications. First, it shows that Rd is increased under the following circumstances: reduced regional blood flow (*Q*), high first-pass regional extraction (*E*) or a high BUI, or a high systemic clearance CL_TB_. Thus, it shows the disadvantage of applying IA therapies to treating brain disorders in which the blood flow is 50 mL/100 g/min, with the BBB generally preventing drug uptake. Second, the model shows that IA drug delivery will be useful for a drug that has high regional extraction (*K*_in_ or BUI), if the blood flow to the brain can be safely decreased or if the systemic clearance of the drug is low. However, this model has several inherent limitations due to assumptions made. First, the model assumes that there is uniform mixing of drug in the arterial blood, thereby ignoring regional variations in drug concentrations due to streaming. A second assumption is that there is no drug efflux and therefore highly diffusible molecules may have a rapid uptake and high first-pass extraction, but will rapidly wash out once the arterial concentrations decline. Moreover, the model also assumes homogeneous retention of drugs by the brain tissue, which is not always the case. Highly diffusible tracers and drugs are likely to be washed out directly proportional to regional blood flow. Despite these concerns, the model is very useful in understanding the applications of IA drugs. This model was also compartmentalized into tumor and normal tissue and by varying the relative blood flow in these two compartments the model could then be altered to investigate how blood flow and tumor drug delivery could be improved.

### 6.2. Computational Fluid Dynamic Models of Regional Drug Delivery

With the advent of nanotechnology, much greater emphasis is being placed on developing models of drug delivery that include fluid dynamic factors. The uptake of the nanoparticles is described as a function of the probability of adhesion. Hossain et al. provide an accurate description of regional drug delivery of nanoparticles in the coronary circulation [10]. This model uses human MR data to acquire vascular dimensions and then applies isogeometric corrections to the dimensions of the vascular lumen. It assumes Newtonian properties of blood. It describes the movement of nanoparticles on the parabolic wave front as they are presented to the vascular endothelium. The probability of adhesion of a nanoparticle in this model is determined by several key factors including the number of nanoparticles released, vascular geometry, input flow function, and the size and shape of the nanoparticles. The flow function, along with the properties of the nanoparticles such as the size and the shape, determines hydrodynamic and shear stress that tend to dislodge the particles from the vascular endothelium. Other intrinsic properties of the nanoparticle, such as ligand density of the nanoparticle and the density of receptors on the endothelium, determine the likelihood of particles binding to the endothelial surface. The net uptake of the nanoparticles is a balance of two opposing forces: the force with which the particle attaches to the endothelium and the hydrodynamic force that tends to dislodge it. The model was tested* in vitro* to determine the effects of shear forces and particle size. The experimental data was consistent with the model predictions. A similar model describing nanoparticle delivery reported by Liu et al. revealed how the shape of the particles, for a given particle volume, further affects the probability of adhesion [86].

**Figure 6:**
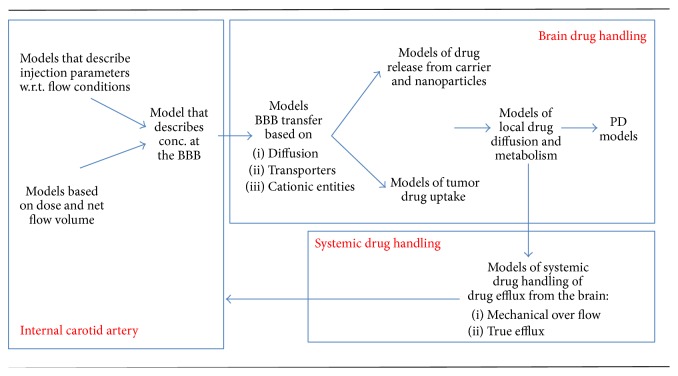
Integrated framework for characterizing intra-arterial delivery models.

The Hossain and Liu et al. models are limited to the description of particle delivery to the vascular endothelial surface. These models describe IV delivery. However, these models provide valuable insight into the hydrodynamic factors that will, probably even more profoundly, affect IA drug delivery. Older models of IA drug delivery completely overlooked the hydrodynamic factors. As of yet, there is no model taking into account all aspects of IA drug delivery. Many models applying limited components of IA drug delivery are currently available and are being applied in the clinical setting. Attempts are currently underway to integrate several of these models into a composite descriptor of IA drug delivery (Figure 6) [10, 86].

## 7. Recent Studies

The pharmacological rationale for IA chemotherapy has not changed significantly over the past five decades. The scope of IA therapies remains that of targeted drug delivery. There have been numerous improvements that could have bearing on modern IA chemotherapy trials. Several important advancements in technology and patient selection have significantly changed the landscape of IA drug delivery.  Table 4 summarizes the most recent published clinical trials employing IA chemotherapy for GBM.

### 7.1. Patient Selection

With increased experience and availability of new biomarkers defining patient specific characteristics, tailored therapeutic strategies will certainly be the modus operandi in the not so distant future. Genetic markers, such as MGMT gene expression, could further aid in selecting those patients that would benefit most from IA temozolomide [117, 118]. Using appropriate adjuvant measures in conjunction with careful patient selection could enhance the safety and efficacy of IA treatments. Although IA GBM treatment has shown relatively little clinical benefit thus far, the good results seen with primary central nervous system lymphomas, low-grade astrocytomas, and oligodendrogliomas give hope.

### 7.2. Catheter Technology

In the early years, polyethylene catheters were surgically implanted to deliver drugs, such that infusions were performed over a period of several days. Hemorrhage, thrombosis, and vasospasm were reported with such placement. As expected, catheter technology has significantly improved over the years. Drugs can now be delivered superselectively, above the ophthalmic artery, thereby minimizing ocular complications. Thrombotic catheter complications can also be mitigated by newly developed anticoagulant/antiplatelet drugs [119, 120].

### 7.3. Imaging Technology

A major advancement that could impact IA chemotherapy is the capability to assess regional blood flow from angiographic transit time [121]. Ability to assess blood flow could prove extremely valuable in determining the injection profile of chemotherapeutic drugs in order to enhance safety and efficacy of the procedure.

### 7.4. Drug Selection

Due to its high lipid solubility, short biological half-life, and rapid onset of action, nitrogen mustard compounds (BCNU, ACNU, and HeCNU) have been investigated in past clinical trials. These compounds have had significant neurological complications. Recent studies have therefore focused on drugs with reduced local toxicity and are therefore safer for IA delivery, such as carboplatin and methotrexate.

## 8. New Paradigms in IA Chemotherapy

Several key points have led to a paradigm shift in IA chemotherapy for GBM in recent years. The emphasis has shifted from traditional chemotherapeutic compounds to relatively nontoxic biological interventions. The availability of biological agents, such as the VEGF antagonist bevacizumab, has led to a considerable increase in the safety profile of IA chemotherapy [17]. However, the impact of such drugs on survival is still debatable. On the other hand, new biological compounds and improved IA delivery methods could potentially have a tremendous impact on the efficacy and safety of IA treatments. For example, flow arrest during IA delivery of drugs has several important benefits including better targeting of the drugs to the tumor site, achieving higher cerebral arterial concentrations, achieving more consistent concentrations in the arterial distribution, and increased transit time with decreased shear stress and avoidance of binding of drugs to blood proteins or other cellular elements. The Dedrick model shows the importance of blood flow in influencing the kinetics of IA drugs. Reduction of blood flow has been used to augment the effects of drugs in the IA treatment employed for liver and breast cancer. The brain can safely tolerate up to three minutes of ischemia in healthy normothermic individuals. Therefore, we strongly advocate for flow arrest during IA chemotherapy for GBM, as an essential part of optimizing drug delivery to brain tumors.

Improvements in IA drug delivery can be further enhanced by developing better drug delivery protocols that account for anatomic and physiologic variables. In preclinical research, this can be done by optically monitoring and correcting for the variable degree of BBB disruption and, ultimately, monitoring the response of brain tissue during treatment. Clinically safe and reversible means of decreasing cerebral blood flow, such as hyperventilation, hypothermia, or deep anesthesia, could be used to improve drug delivery.

Another important component is the design of drugs specifically tailored for IA delivery.

The conventional design of drugs relies on pharmacokinetic properties of compounds that result in preferential uptake by the target site. However, if a drug is directly delivered to the target site by IA injections, the emphasis on such characteristics becomes redundant. Classical preclinical screening of drug isomers does not adequately select for IA use; drug isomers and formulations for effective IA delivery are likely fundamentally different from those administered systemically. For IA delivery, drug design has to ensure maximum tumor uptake and adequate retention of drugs, in order to achieve the desired therapeutic effects while avoiding regional toxicity. The uptake has to be rapid, virtually within the transit time through the capillary network. The problem of drug delivery is further compounded by the presence of the BBB that severely restricts the uptake of many therapeutic compounds.

Specific methods can be used to improve IA drug design in order to augment delivery. Small molecule chemotherapy (<400 Daltons) significantly facilitates uptake across the BBB [122]. Increasing lipid solubility, either by adding methyl groups, replacing polar groups, or adding halogenated alkane chains, such as tributyl chlorambucil, further increases diffusivity [123]. Alternatively, using liposomal carrier systems optimized for size and surface charge with rapid drug offloading characteristics may be effective [124]. Furthermore, another novel alternative is immunoliposomes, nonspecifically targeted to the vascular endothelium (CD 31, ACE, or Factor VIII/vWF-Ag), to the transport systems (OX-26 antibody to transferrin receptor), or to the pathology specific antigens (ICAMs) [125–128]. Immunoconjugated drugs, such as OX26-methotrexate and OX26-daunorubicin, chimeric drugs using high capacity transporter systems, or drugs with exceedingly brief duration of actions that are hydrolyzed during transit through cerebral/regional circulation by ubiquitous enzymes, such as esterases or alkaline phosphatases, are also intriguing options that should be considered for IA delivery [128].

Osmotic disruption of the BBB is often employed to augment IA drug delivery. Although the blood brain barrier is already compromised around the tumor bed, disruption of the BBB further improves drug permeability and tumor response. Alternatives to mannitol are being actively developed but are not yet available in the clinical setting. Bradykinin analogues have been a promising alternative but have given way to focused ultrasound in recent years [14, 129, 130]. Perhaps a better alternative to BBB disruption might be the use of cell-penetrating peptides [131]. There is some evidence that IA delivery of cell-penetrating peptides can penetrate the BBB and preliminary results suggest that they can lead to tumor specific drug uptake. Similarly, OX-26 antitransferrin receptor antibody also targets the vascular endothelium of the tumor cells after IA injections. Both anionic ligands and transferrin receptors are relatively overexpressed in the tumor tissue compared to normal brain and they provide convenient targets for tumor-specific drug delivery [132]. These preliminary results suggest that it might be possible to bypass the BBB and achieve tumor-selective drug delivery.

Temozolomide (TMZ) is currently the first-line drug for GBM treatment after surgery. It is a relatively small molecule capable of easily diffusing across the BBB [133]. To that end, increased permeability is an important advantage for systemic chemotherapy. TMZ also acts synergistically with radiation, with both being part of a combined treatment regimen. However, despite its theoretical advantages, TMZ also provides a good example of the shortcomings of systemic chemotherapy. Systemic administration can only achieve 10% of the observed* in vitro* tumoricidal concentrations. Furthermore, currently prescribed doses of TMZ have been demonstrated to cause immunosuppression. Gliomas are notorious for suppressing the local immune response. Therefore, systemic TMZ may aggravate this immunosuppressive effect, further affecting outcomes [25].

To overcome the problem of systemic drug delivery, a concerted effort is now underway to develop nanoparticles that could be injected either systemically, reaching the tumor site due to the intrinsic homing capabilities, or via novel routes such as IA or intranasally. Yet, complex theoretical and practical hurdles to delivery remain. Increased capillary permeability lowers the diffusion barrier while also creating a fluid flux away from the lesion. In addition, within the tumor mass, there are areas of necrosis and hemorrhage, with capillary networks compressed by malignant cells. The tumor stroma also poses a barrier to drug diffusion, particularly in the case of larger nanoparticles. Therefore, full tumor penetration of drugs is extremely difficult, irrespective of delivery method. The microscopic tumor characteristics will hinder complete eradication of tumor cells through purely chemotherapeutic means, with certain populations of malignant cells remaining beyond the reach of drugs.

## 9. Conclusion

Given the complexity of drug delivery to the brain, conventional pharmacokinetic approaches cannot be applied to the delivery of large and complex molecules, genes, and peptides. Hence, innovative approaches, such as intra-arterial delivery, have to be considered to ensure effective tumor targeting of such novel pharmaceuticals. GBM, in particular, requires a coherent treatment strategy simultaneously targeting multiple mechanisms of tumorigenesis and progression. IA delivery is a well-conceived strategy for delivering such agents that may prove to be an integral component of the protocol for combating this uniformly fatal disease.

## Figures and Tables

**Table 1:** Methods of targeting drugs to glioblastoma.

	Example agent	FDA approved	Advantages	Disadvantages
Oral	Temozolomide	Yes	Noninvasive administration	Systemic toxicity, myelosuppression

Intravenous	Bevacizumab	Yes	Minimally invasive administration	Systemic toxicity, CNS hemorrhage, and thromboembolic events

Local polymers	Carmustine implant (Gliadel)	Yes	Delivery directly to tumor resection bed	Craniotomy for implantation required, small volume of drug distribution, and relying on diffusion, seizure, and infection

Intra-arterial	Bevacizumab	No	Minimally invasive superselective delivery to tumor feeding arteries	High first-pass drug extraction necessary

Intraventricular	Methotrexate	No	Ideal for intraventricular and leptomeningeal disease	Neurotoxicity, aseptic meningitis, need for ventricular access device, and limited value for parenchymal tumor

Intrathecal	Methotrexate	No	Ideal for intraspinal and leptomeningeal disease	Neurotoxicity, aseptic meningitis, need for lumbar infusion, and limited value for parenchymal tumor

Microdialysis	Methotrexate	No	Limiting systemic and neurotoxicity, tissue delivery, and sampling possible	Small volume of drug distribution, relying on diffusion

Convection-enhanced	Topotecan	No	Limiting systemic and neurotoxicity, diffusion independent, and continuous infusion with implantable pumps possible	Surgical implantation required

Intranasal	Perillyl alcohol	No	Noninvasive administration	Unpredictable targeting and volume of distribution, mucosal irritation

**Table 2:** Recent clinical trials employing IA chemotherapy for CNS tumors.

Trial	Drugs	Tumor type
NCT01811498	Bevacizumab	GBM
NCT01238237	Cetuximab	GBM, anaplastic astrocytoma
NCT01180816	Temozolomide	GBM, anaplastic astrocytoma
NCT01269853	Bevacizumab	GBM, anaplastic astrocytoma
NCT00075387	Carboplatin-based chemotherapy	High grade glioma
NCT01083966	Bevacizumab	Vestibular schwannoma
NCT00253721	Melphalan	Brain and CNS tumors, lymphoma, and metastatic cancer
NCT01386710	Bevacizumab, Carboplatin	GBM, anaplastic astrocytoma
NCT00983398	Melphalan, Carboplatin, Sodium Thiosulfate, Filgrastim, and Pegfilgrastim	CNS embryonal tumors and germ cell tumors
NCT02285959	Bevacizumab	GBM
NCT01884740	Erbitux, Bevacizumab	GBM and other tumors

**Table 3:** Optical methods employed to study IA drug pharmacokinetics.

Method	Depth	Advantage	Disadvantage
Diffuse reflectance spectroscopy	1-2 mm	(i) Insensitive to scattering changes (ii) Low cost (iii) High spectral resolution	(i) Recovery of relative absorption (ii) Low spatial resolution

Diffuse optical tomography	up to ~4 cm	3D reconstruction of absolute optical pharmacokinetics (OP)	(i) Expensive (ii) Bulky (iii) Low spectral resolution

Frequency domain photon migration	up to ~4 cm	Recovery of absolute OP	(i) Low spectral resolution (ii) Expensive (iii) Low spatial resolution (iv) Not suitable for small geometries

Diffuse optical spectroscopic imaging	up to ~4 cm	(i) 3D reconstruction of absolute OP (ii) High spectral resolution	(i) Expensive (ii) Not suitable for small geometries

Spatial frequency domain imaging	up to ~1 cm	(i) Noncontact (ii) High spatial resolution (iii) Depth sensitivity	(i) Long acquisition times (ii) Expensive (commercial)

Multispectral imaging	up to ~1 cm	(i) Low cost (ii) High spatiotemporal acquisition rate	(i) Relative absorption values (ii) Susceptible to scattering effects (iii) No depth resolution

**Table 4:** Studies using intra-arterial chemotherapy for brain tumors.

Author, year	Drugs	Pathology
Fortin et al., 2014 [20]	Carboplatin and Melphalan	Recurrent GBM
Jeon et al., 2012 [23]	Bevacizumab	Recurrent GBM
Shin et al., 2012 [25]	Bevacizumab, Temozolomide, and Cetuximab	Recurrent GBM
Boockvar et al., 2011 [17]	Bevacizumab	Recurrent GBM
Imbesi et al., 2006 [22]	Nimustine (ACNU)	Newly diagnosed GBM
Hall et al., 2006 [21]	Carboplatin, Methotrexate	Recurrent pontine GBM
Fortin et al., 2005 [19]	Carboplatin, Methotrexate	GBM and other tumors
Qureshi et al., 2001 [24]	Carboplatin + Cereport	GBM and other tumors
Gobin et al., 2001 [14]	Carboplatin + Cereport	GBM and other tumors
Chow et al., 2000 [18]	Carboplatin + Cereport	Recurrent GBM
